# Folate receptor 1 is a stemness trait-associated diagnostic and prognostic marker for hepatocellular carcinoma

**DOI:** 10.1186/s40364-025-00752-8

**Published:** 2025-03-04

**Authors:** Yuto Shiode, Takahiro Kodama, Yu Sato, Ryo Takahashi, Takayuki Matsumae, Kumiko Shirai, Akira Doi, Yuki Tahata, Hayato Hikita, Tomohide Tatsumi, Moto Fukai, Akinobu Taketomi, Mathuros Ruchirawat, Xin Wei Wang, Tetsuo Takehara

**Affiliations:** 1 Department of Gastroenterology and Hepatology, Osaka University, Graduate School of Medicine, 2-2 Yamadaoka Suita, Osaka, 565-0871 Japan; 2 Laboratory of Human Carcinogenesis, Center for Cancer Research, National Cancer Institute, Bethesda, MD USA; 3 Department of Gastroenterological Surgery I, Graduate School of Medicine, Hokkaido University, Sapporo, Japan; 4 Laboratory of Chemical Carcinogenesis, Chulabhorn Research Institute, Bangkok, 10210 Thailand; 5 Center of Excellence On Environmental Health and Toxicology (EHT), OPS, MHESI, Bangkok, Thailand; 6 Liver Cancer Program, Center for Cancer Research, National Cancer Institute, Bethesda, MD USA

**Keywords:** HCC, FOLR1, Biomarker, Diagnosis, Prognosis

## Abstract

**Background:**

Hepatocellular carcinoma (HCC) can be classified into several subtypes based on molecular traits, aiding in prognostic stratification. The subtype with a poor prognosis is often associated with stem/progenitor features. This study focused on identifying circulating biomarkers for aggressive HCC.

**Methods:**

We searched for secretory proteins whose expression was positively associated with the stem/progenitor markers KRT19, EPCAM, and PROM1 in 2 independent HCC cohorts. Serum folate receptor 1 (FOLR1) levels were measured in 238 chronic liver disease and 247 HCC patients, evaluating their diagnostic and prognostic capabilities.

**Results:**

FOLR1 was identified as a secretory protein that was positively correlated with all 3 stem/progenitor markers and a poor prognosis in both the discovery and validation cohorts. Higher FOLR1 expression was detected in tumor than nontumor tissues and was associated with aggressive subtypes, and activation of p53, DNA repair, Myc, E2F, and PI3K/AKT/mTOR pathways. Serum FOLR1 levels correlated with tumoral FOLR1 expression in HCC patients and were significantly elevated compared with those in patients with chronic hepatitis or nonliver disease. Serum FOLR1 levels demonstrated diagnostic performance for HCC comparable to that of alpha-fetoprotein (AFP), and their combination increased the diagnostic accuracy. Elevated serum FOLR1 levels were associated with poor prognosis in HCC patients, regardless of treatment, especially in patients with early-stage disease. The multivariate analysis revealed that the serum FOLR1 level and the Gender, Age, AFP-L3, AFP, and Des-gamma-carboxy prothrombin (GALAD) score were independent predictors of a poor prognosis with their combination further stratifying prognosis.

**Conclusions:**

FOLR1 is a stemness-associated biomarker for HCC, with serum levels serving as a diagnostic marker for HCC and a prognostic indicator for early-stage disease.

**Supplementary Information:**

The online version contains supplementary material available at 10.1186/s40364-025-00752-8.

## Background

Primary liver cancer (PLC) remains a global health challenge, with an estimated incidence of > 1 million cases by 2025 [1], and is the sixth most common malignancy worldwide [2]. Hepatocellular carcinoma (HCC) is the major form of PLC and accounts for approximately 80% of cases [3]. Despite advancements in diagnostic and treatment modalities, the prognosis of HCC remains a significant concern [4]. The 5-year survival rate for HCC patients remains alarmingly low, except for those diagnosed at an early stage, with rates of 54.5%, 29.2%, 9.8%, and 4.0% for stages A, B, C, and D, respectively, as classified by the BCLC system [5, 6]. Current diagnostic methods for HCC primarily rely on imaging, serum biomarkers, and histopathological assessments [7]. The complex etiology and diverse molecular subtypes of HCC pose challenges in predicting patient prognosis during diagnosis and tailoring effective treatment strategies [1, 8–11]. Precise markers that can enhance the prognostic prediction are urgently needed to improve patient outcomes.

The molecular classification of HCC has been conducted in the past via microarray technology and, more recently, by RNA/DNA sequencing using next-generation sequencers [12, 13]. Several classifications have been proposed, including 3 clusters (S1–3) by Hosida [14], 6 clusters by Boyault [8], 4 clusters by Chiang [15], 3 clusters by Murai [16], and the most recent 3 clusters by The Cancer Genome Atlas (TCGA) [17]. These classifications are particularly useful for stratifying patients according to prognosis. In these classifications, some subclasses are unique and/or different from one classification to another, suggesting the substantial molecular heterogeneity of HCC [12, 18]. Importantly, all the classifications identified a common subclass with an aggressive phenotype and poor prognosis named the proliferation class by Chiang, G1 by Boyault, S2 by Hoshida, and Cluster 1 by TCGA [12]. This subclass is characterized by chromosomal instability, global DNA hypomethylation, and especially stem cell phenotypes, with increased expression of stem/progenitor markers such as KRT19, EPCAM, and PROM1 [12, 19]. Indeed, all these stem/progenitor markers are associated with a poor prognosis and aggressive behavior of HCC [19–22]. Identifying such a poor prognostic subclass may help to determine better treatment selection and disease monitoring but requires invasive tumor biopsy. Because HCC is accurately diagnosed by contrast-enhanced computed tomography (CT) or Magnetic Resonance Imaging (MRI) without tumor biopsy in daily practice, the development of noninvasive biomarkers is highly desirable.

In this study, we hypothesized that secretory proteins, whose expression is associated with hepatic stem/progenitor markers in HCC, may serve as blood-based prognostic biomarkers for HCC. First, we analyzed RNA-sequencing data from 2 independent large-scale HCC cohorts. We found that folate receptor 1 (FOLR1), a known secretory protein [23], was upregulated in tumor tissues and positively associated with 3 major stem/progenitor markers, KRT19, EPCAM, and PROM1 [19, 24, 25], and a poor prognosis for HCC patients in both the discovery and validation cohorts. We found that circulating FOLR1 levels were positively correlated with FOLR1 tumoral levels in HCC patients. We subsequently proved that serum FOLR1 levels can serve as a novel biomarker for HCC detection and prognostic predictions of early-stage HCC, suggesting potential utility for improved risk stratification and personalized treatment strategies.

## Methods

### Acquisition and analysis of the discovery cohort from TCGA data

The RNA-seq data registered in The Cancer Genome Atlas (TCGA) were downloaded from FIREHOSE (https://gdac.broadinstitute.org/) and analyzed with Gene Pattern (https://www.genepattern.org/). Secretory proteins were extracted from the Human Protein Atlas (https://www.proteinatlas.org/humanproteome/tissue/secretome). For candidate gene selection, we first searched for secretory proteins whose expression was strongly associated with the expression of all 3 stem/progenitor markers, including KRT19, EPCAM, and PROM1 (correlation coefficient ≥ 0.35). Then, we extracted genes that were highly upregulated in tumor tissues compared with nontumor tissues. We further selected genes predicted to encode secretory proteins based on a database in the Human Protein Atlas. Finally, we selected genes whose high expression was positively associated with a poor patient prognosis (*p* ≤ 0.05). A nearest template prediction (NTP) analysis was used to determine the various molecular classifications of each patient registered in TCGA. GSEA with DESERT_STEM_CELL_HEPATOCELLULAR_CARCINOMA_SUBCLASS_UP and ssGSEA with hallmark gene sets were performed to determine the biological state of each case registered in TCGA.

### Acquisition and analysis of the validation cohort from TIGER-LC data

The validation cohort data used in this study were derived from the the Thailand Initiative in Genomics and Expression Research for Liver Cancer (TIGER-LC) consortium, comprising 50 patients diagnosed with hepatocellular carcinoma (HCC). The TIGER-LC cohort included a comprehensive set of paired surgical tumor and nontumor samples from sequential patients with liver cancer across five major hospitals in Thailand: Maharaj Nakorn Chiang Mai Hospital, Roi Et Hospital, Chulabhorn Hospital-Bangkok, the National Cancer Institute of Thailand, and Srinagarind Hospital. Recruitment focused on patients with confirmed diagnoses of primary liver cancers (HCC and intrahepatic cholangiocarcinoma (ICC)), as well as high-risk patients and healthy controls. The diagnosis of HCC was based on physician assessments using criteria such as elevated serum AFP levels, ultrasound imaging, and/or histopathological examinations. Samples were collected sequentially from each hospital’s weekly schedule of liver cancer surgeries and patient referrals, ensuring the representative inclusion of patients with recent HCC diagnoses. Patients with mixed HCC-ICC were excluded from this study. Clinical, demographic, socioeconomic, and morbidity data were collected through comprehensive questionnaires and medical records. Institutional review board (IRB) approval was obtained from each participating center (Maharaj Nakorn Chiang Mai Hospital, Roi Et Hospital, Chulabhorn Hospital-Bangkok, National Cancer Institute of Thailand, and Srinagarind Hospital), with all participants providing written informed consent. The exclusion criteria for the study population included individuals under 20 or over 80 years of age, those diagnosed with HIV, residents in institutional settings, and those who were severely ill at recruitment.

### Analysis of patients’ serum samples

This retrospective cohort study enrolled 503 patients who were admitted to Osaka University Hospital between 2014 and 2018 (238 patients with chronic hepatitis, 215 patients with HCC, and 50 patients with colorectal polyps) and 32 HCC patients who underwent surgical resection at Hokkaido University Hospital between 2007 and 2018. A diagnosis of HCC was made by liver imaging tests (computed tomography (CT) and Magnetic Resonance Imaging (MRI)). All patients provided informed consent, and the study design was consistent with the principles of the Declaration of Helsinki. The protocol for the study involving patient serum and tissues was approved by the Institutional Review Board Committee of Osaka University Hospital (Institutional Review Board No. 17097).

### RNA isolation and qPCR

Total RNA was extracted from human liver tissues using the RNeasy Mini Kit (Qiagen, Venlo, Netherlands) according to the manufacturer's protocol. RNA concentration and purity were assessed using a NanoDrop 2000 spectrophotometer (Thermo Fisher Scientific, Waltham, MA), and samples with an A260/A280 ratio of 1.8–2.1 were used for subsequent analyses. One microgram of total RNA was reverse transcribed into complementary DNA (cDNA) using the ReverTra Ace qPCR RT Kit (Toyobo, Tokyo, Japan) following the manufacturer's instructions. Quantitative real-time PCR (qPCR) was performed using the THUNDERBIRD Probe qPCR Mix (QPS101; Toyobo, Osaka, Japan) on a QuantStudio 7 Real-Time PCR System (Thermo Fisher Scientific). Each qPCR reaction was carried out in a 20 μL reaction volume containing 10 μL of THUNDERBIRD Probe qPCR Mix, 1 μL of cDNA, 900 nM of each forward and reverse primer, 250 nM of TaqMan probe, and nuclease-free water. The TaqMan gene expression assays used were for GAPDH (Hs02786624_g1) and FOLR1 (Hs06631528_s1). The thermal cycling conditions were as follows: initial denaturation at 95 °C for 30 s, followed by 40 cycles of 95 °C for 15 s and 60 °C for 1 min. Relative gene expression levels were calculated using the ΔΔCt method, with GAPDH as the reference gene.

### ELISAs

The preoperative plasma of patients was stored in a − 80 °C deep freezer and analyzed with a Human FOLR1 ELISA Kit (DFLR10, R&D Systems) according to the manufacturer’s protocol.

### Statistical analysis

Statistical analyses were performed using Prism v.10.2.2 for Mac (GraphPad, San Diego, CA; research resource identifier [RRID] SCR_002798), JMP version 14 (SAS Institute, Inc., Cary, NC; RRID SCR_014242), and EZR version 1.65 (Jichi Medical University Saitama Medical Center). Continuous variables were summarized as means ± SDs or medians with interquartile ranges, as appropriate. Differences in continuous variables between two groups were tested using an unpaired two-tailed t test for normally distributed variables and the Mann–Whitney U test for non-normally distributed variables. Comparisons among three groups were performed using analysis of variance (ANOVA) with Tukey's post hoc test. Survival analysis was conducted using the Kaplan–Meier method, and differences between survival curves were evaluated using the log-rank test. The required sample size for survival analysis was calculated using G*Power 3.1 (Heinrich-Heine-Universität Düsseldorf, Germany) with the following parameters: two-tailed test, α = 0.05, power = 0.8, and proportions p1 = 0.5 and p2 = 0.8. The calculation indicated that a total sample size of 20 participants (10 per group) was required to achieve adequate statistical power. For diagnostic accuracy, the DeLong test was used to compare the areas under the ROC curves. Multivariable analyses were performed using Cox proportional hazards models to adjust for potential confounders, including age, sex, and tumor stage, with results expressed as hazard ratios and 95% confidence intervals. For all statistical tests, a *p*-value of ≤ 0.05 was considered statistically significant. The handling of missing data was performed using a complete-case analysis approach unless otherwise stated. Detailed statistical methods and software tools are reported in the CTAT table.

## Results

### Tumoral FOLR1 is a stemness trait-associated prognostic marker for HCC

**Fig. 1:**
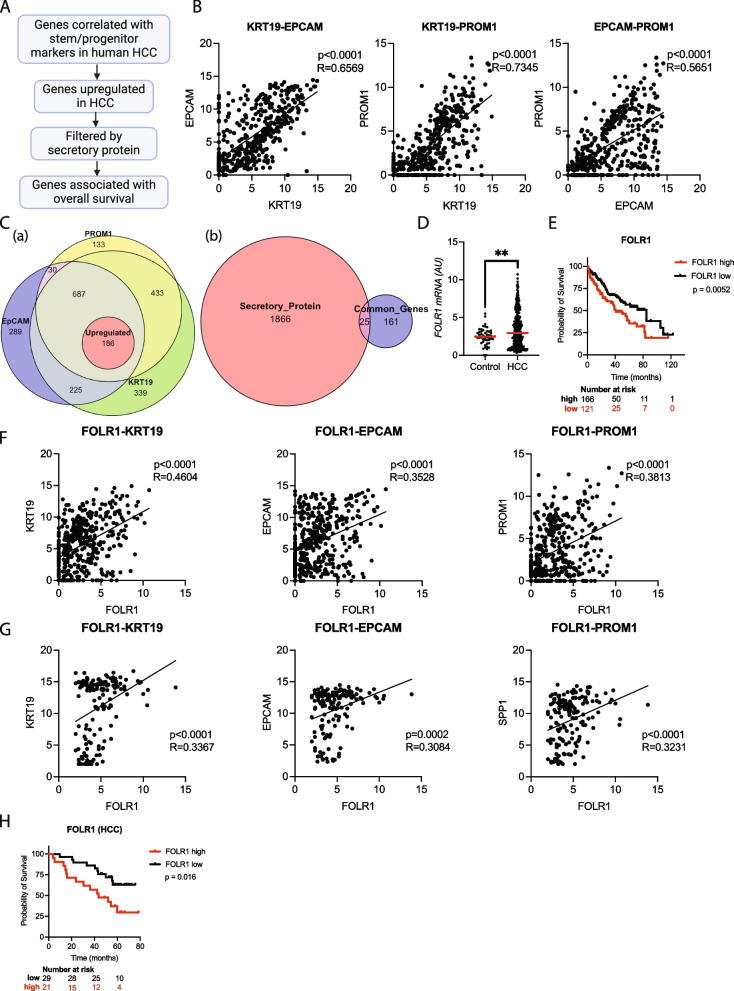
FOLR1 is a tumor-derived and stemness trait-associated prognostic marker for HCC. **(A)** Schematic of the workflow used to identify candidate biomarkers. Genes upregulated in hepatocellular carcinoma (HCC) and correlated with cancer stemness markers (KRT19, PROM1 and EPCAM) were compared to identify common genes. These genes were further filtered to identify those encoding secretory proteins that were associated with overall survival (OS) (created with Biorender). **(B)** Scatter plot showing the correlations between KRT19 and EPCAM **(a)**, KRT19 and PROM1 **(b),** and EPCAM and PROM1 **(c)** mRNA levels in TCGA-LIHC cohort. **(C)** (a) Venn diagram showing the overlap among genes correlated with KRT19 (1870 genes), PROM1 (1469 genes), EPCAM (1417 genes) and upregulated genes (186 genes). (b) Venn diagram showing the overlap among common genes (873 genes) and predicted secretory proteins (186 genes). **(D)** Quantitative PCR analysis showing the expression levels of the folate receptor 1 (FOLR1) mRNA in control and HCC samples from TCGA-LIHC cohort. **p < 0.01. **(E)** Kaplan‒Meier survival curves for TCGA-LIHC cohort stratified by high (red line) and low (black line) FOLR1 mRNA expression levels. **(F-G)** Scatter plot showing the correlation between FOLR1 mRNA expression and KRT19 (left panel), EPCAM (middle panel), and PROM1 (right panel) mRNA expression levels in TCGA-LIHC cohort **(F)** and the Thailand Initiative in Genomics and Expression Research for Liver Cancer (TIGER-LC) cohort **(G)**. (**H**) Kaplan‒Meier survival curves for the TIGER-LC cohort stratified by high (red line) and low (black line) FOLR1 mRNA expression levels

We formulated a comprehensive strategy to identify secretory proteins whose expression is closely associated with the expression of HCC stem/progenitor markers and a poor prognosis as potential blood-based biomarkers for aggressive HCC (Fig. 1A, Supplementary Fig. 1). We first analyzed RNA sequencing data from the HCC cohort in TCGA database. We focused on 3 stem/progenitor markers, KRT19, EPCAM, and PROM, which are known to be associated with tumor aggressiveness. These genes exhibited strong positive correlations with each other (Fig. 1B), confirming that they reflect common stemness features in HCC. We identified 186 genes, expressions of which were positively associated with that of all 3 genes in tumor tissues and were significantly upregulated in tumor tissues compared with nontumor tissues (Fig. 1C left panel, D). Among them, 25 genes were predicted to encode secretory proteins based on a database in the Human Protein Atlas (Fig. 1C right panel, Supplementary Table 1). After analyzing their associations with overall survival (OS), FOLR1 emerged as the only gene with a significant impact on the prognosis (Fig. 1E). We analyzed RNA-sequencing data for tumor tissues from HCC patients registered in the Thailand Initiative in Genomics and Expression Research for Liver Cancer (TIGER-LC) cohorts to externally validate our findings [26]. All 3 stem/progenitor markers were positively correlated with each other (Supplementary Fig. 2) and with FOLR1 (Fig. 1G). Patients with high FOLR1 expression levels experienced significantly shorter survival than those with low expression levels (Fig. 1H). The consistent findings from 2 independent cohorts indicated that FOLR1 was a tumor-derived prognostic marker for HCC.

### Tumoral FOLR1 expression represents a poor prognostic molecular subtype of HCC with aggressive biological features

**Fig. 2:**
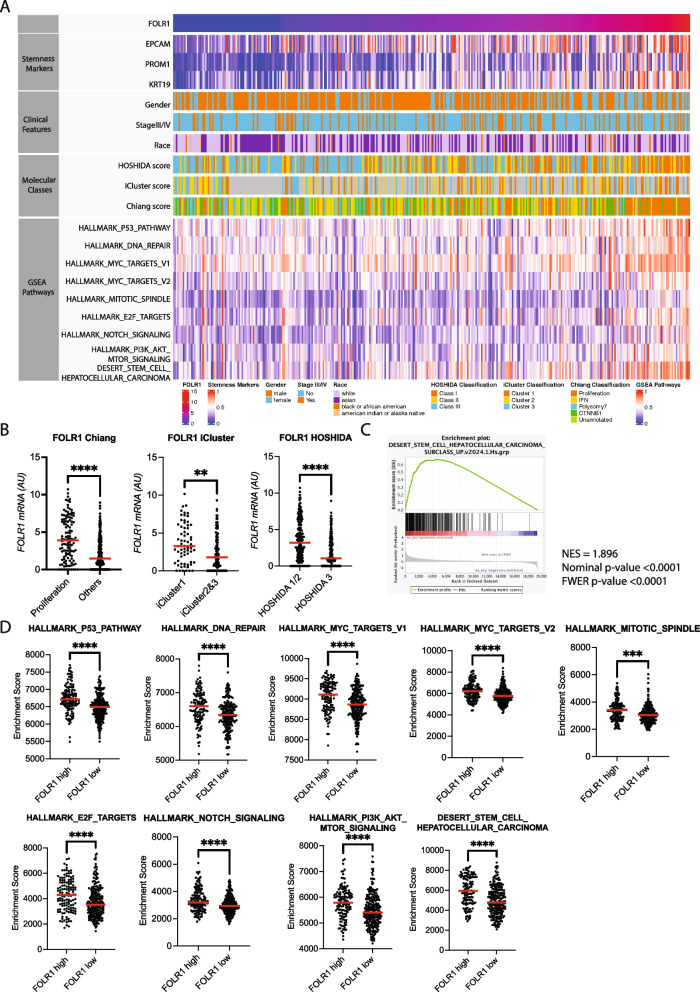
Tumoral FOLR1 expression represents a poor prognostic molecular subtype of HCC with aggressive biological features. **A** Heatmap showing gene expression, clinical features, molecular subclasses, and gene set enrichment scores of hallmark gene sets evaluated by single-set gene set enrichment analysis (ssGSEA). **B** FOLR1 mRNA levels in each molecular class**.** **p < 0.01. ****p < 0.0001.** C** Gene set enrichment analysis (GSEA) comparing the FOLR1 high- and low-expression groups**.** Enrichment plot from GSEA showing the distribution of the enrichment scores for the FOLR1-H and FOLR1-L expression groups.** D** Gene set enrichment scores of HCC patients with either high or low FOLR1 mRNA levels in The Cancer Genome Atlas (TCGA)-LIHC cohort. ****p < 0.0001

We then investigated the molecular characteristics of HCC patients with high FOLR1 expression. We first determined various molecular subclasses for each sample in TCGA cohort. FOLR1 expression levels were significantly higher in the proliferation class in the Chang classification, Cluster 1 in the iCluster classification, and S1/2 in the Hoshida classification (Fig. 2A, B), suggesting that FOLR1 was associated with the poor prognostic subtype with biological aggressiveness and stem/progenitor features. Gene set enrichment analysis (GSEA) revealed the significant upregulation of a pathway linked to HCC stem cell signatures in the high FOLR1 subgroup (Fig. 2A, C). Moreover, single-set GSEA revealed that a variety of oncogenic pathways related to aggressive biological features, including the p53, DNA repair, MYC, E2F, and PI3K/AKT/MTOR pathways, were activated in the high-FOLR1 subgroup (Fig. 2A, D). Overall, tumoral FOLR1 expression represents a poor prognostic molecular subtype of HCC with aggressive biological features.

### The serum FOLR1 level is a diagnostic biomarker of HCC, especially in combination with AFP

**Fig. 3:**
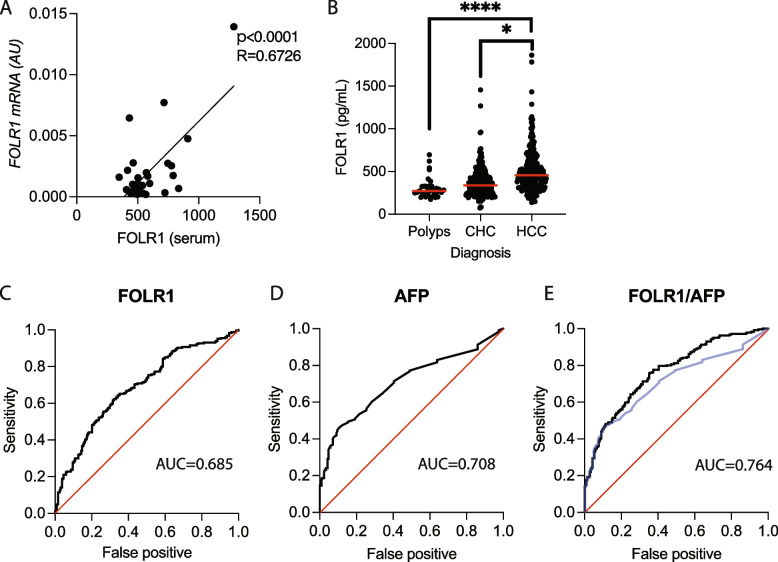
The serum FOLR1 level is a diagnostic biomarker of HCC, especially in combination with AFP. **A** Scatter plot showing the correlation between FOLR1 mRNA expression and serum FOLR1 levels in the surgically resected HCC cohort. **B** Comparison of serum FOLR1 levels among patients with colorectal polyps, patients with chronic hepatitis C (CHC), and hepatocellular carcinoma (HCC) patients. *p < 0.05. ****p < 0.0001. **C-D** Receiver operating characteristic (ROC) curves representing the diagnostic performance of FOLR1 (**C**) and alpha-fetoprotein (AFP) (**D**) for HCC. **(E)** ROC curve illustrating the combined diagnostic performance of FOLR1 and AFP for HCC. The black line represents the FOLR/AFP combination, and the blue line represents AFP

FOLR1 was selected based on its secretory potential, and we thus examined whether tumor FOLR1 levels are reflected peripherally in HCC patients. To this end, we measured the serum FOLR1 levels and tumor FOLR1 expression levels in 32 HCC patients who underwent surgical resection. We detected a significant positive correlation between these parameters (Fig. 3A), suggesting that serum FOLR1 levels may reflect tumoral FOLR1 expression in HCC patients. Therefore, we next pursued the potential of FOLR1 as a blood-based biomarker for HCC patients by evaluating the serum of 247 patients diagnosed with HCC and 238 patients diagnosed with chronic hepatitis C (CHC) without HCC, along with 50 patients diagnosed with colon polyps without liver disease who served as normal controls. Compared with CHC patients, HCC patients were significantly older, predominantly male, and presented higher fibrosis-4 (FIB-4) index values, while their platelet counts, prothrombin times, and albumin levels were significantly lower (Table 1). Compared with those in both normal controls and CHC patients, serum FOLR1 levels were significantly elevated in HCC patients (Fig. 3B). The univariate logistic regression analysis revealed that FOLR1 levels, albumin–bilirubin (ALBI) scores, the FIB-4 index, alpha-fetoprotein (AFP), alanine aminotransferase (ALT), g-GTP, alkaline phosphatase (ALP), and albumin levels, the platelet count, age, and sex were associated with the occurrence of HCC among CHC patients (Table 2). The multivariate analysis revealed FOLR1 levels, AFP levels, ALT levels, ALP levels, age and sex as independent diagnostic factors for HCC in CHC patients (Table 2). The serum FOLR1 level discriminated between HCC and CHC patients with an area under the receiver operating characteristic curve (AUROC) of 0.685 (Fig. 3C), which was comparable to the AFP level, with an AUROC of 0.708 (Fig. 3D). The optimal threshold level of FOLR1 for indicating a higher diagnostic value for HCC is 409.45 pg/mL. At this threshold, the sensitivity is 61.5% and the specificity is 68.5%. Compared with AFP alone, the combination of these two markers yielded an AUROC of 0.764 for discriminating HCC from CHC, indicating a significant improvement in diagnostic performance (*p* = 0.019) (Fig. 3E). In addition, we have constructed a diagnostic model based on multivariate regression analysis incorporating FOLR1 and other clinical variables. Nomogram-based diagnostic model showed significantly higher diagnostic value compared to AFP or FOLR1 alone (Supplementary Fig. 3). Taken together, these findings indicate that the serum FOLR1 level may be a potential tumor marker for detecting HCC, especially in combination with AFP.

**Table 1:** Background of CHC and HCC patients in our cohort

Factor		All (*N* = 485)			CHC(*N* = 238)		HCC(*N* = 247)		*P* value
	Unit	MEDIAN	IQR	Missing (N)	MEDIAN	IQR	MEDIAN	IQR	
FOLR1	ng/µL	391.38	284.2–514.4	0	337.3	249.6–439.1	455.4	333.8–575.6	< 0.001
Age	Years	71	61–78.5	0	67	55–74	75	68–81	< 0.001
Sex(M/F)		265/220		0	91/147		174/73		< 0.001
BMI	kg/m2	22.6	20.6–25.4	0	22.8	20.5–25.6	22.5	20.6–25.3	0.645
WBC	/mm3	4800	3750–5780	1	4990	4020–5860	4580	3460–5720	0.097
Hb	g/dL	12.95	11.7–14.2	1	13.1	12.1–14.5	12.7	11.2–14	< 0.001
Plt	10^4^/μL	14.5	8.9–19.6	0	16.7	10.825–21.3	13.2	7.9–17.3	< 0.001
AST	U/L	41	29–59.5	0	41.5	29–62	40	30–58	0.200
ALT	U/L	34	22–53	0	39	24–59	29	20–44	< 0.001
ALP	U/L	291.5	226–394.25	3	272	209.5–349.5	323	240.8–461.5	< 0.001
Na	mmol/L	140	139–141	76	140	139–141	140	138–141	0.030
T-Bil	mg/dL	0.7	0.5–0.9	0	0.6	0.5–0.8	0.7	0.5–1	0.019
g-GT	U/L	40	25–70	1	35	23–59.5	46	29–84	0.006
Cr	mg/dL	0.74	0.62–0.88	33	0.68	0.58–0.8025	0.8	0.69–0.95	< 0.001
PT	%	83	72–93	3	87	75.5–95	80	69–90	< 0.001
PT-INR		1.09	1.04–1.17	5	1.07	1.03–1.15	1.11	1.04–1.18	0.347
ALB	g/dL	3.9	3.5–4.2	1	4	3.7–4.3	3.7	3.3–4.1	< 0.001
AFP	ng/mL	7	3–18.5	36	4	3–9	11	5–54.5	0.060
Fib4-index		3.68	2.11–6.61	0	2.81	1.67–5.18	4.57	2.81–7.51	< 0.001
NLR		2.15	1.49–3.25	2	2.02	1.42–2.8	2.41	1.54–3.40	0.333
Child–Pugh	(5/6/7/8/9/10/11/12)	281/79/51/27/7/6/1/1		32	178/29/12/9/1/3/1/1		99/50/39/18/6/3/0/0		< 0.001
ALBI		-2.64	-2.25—-2.91	2	-2.77	-3.02—-2.50	-2.44	-2.79—-2.11	< 0.001

Abbreviations: *Age* Patient Age, *Sex (M/F)* Sex (Male/Female), *BMI* Body Mass Index, *WBC* White Blood Cell count, *Hb* Hemoglobin, *Plt* Platelet, *AST* Aspartate Aminotransferase, *ALT* Alanine Aminotransferase, *ALP* Alkaline Phosphatase, *Na* Sodium, *T-Bil* Total Bilirubin, *γ-GT* Gamma-Glutamyl Transferase, *Cr* Creatinine, *PT* Prothrombin Time, *PT-INR* Prothrombin Time-International Normalized Ratio, *ALB* Albumin, *AFP* Alpha-Fetoprotein, *FIB4-index* Fibrosis-4 Index, *NLR* Neutrophil–Lymphocyte Ratio, *Child–Pugh* Child–Pugh Score, *ALBI* Albumin-Bilirubin Score, *IQR* Interquartile Range

**Table 2:** Logistic Regression Analysis for the dianogosis of HCC in 485 Patients

Factor	Univariate analysis			Multivariate analysis		
	Odds ratio	95% CI	*P* value	Odds ratio	95% CI	*P* value
FOLR1	1.003	1.002–1.005	< 0.001	1.002	1.000–1.003	0.013
NLR	1.045	0.955–1.145	0.329			
ALBI	2.665	1.814–3.916	< 0.001	2.073	0.215–19.994	0.528
Fib4-index	1.123	1.069–1.179	< 0.001	1.085	0.977–1.205	0.120
AFP	1.022	1.013–1.032	< 0.001	1.021	1.010–1.032	< 0.001
BMI	0.990	0.948–1.034	0.644			
AST	0.998	0.994–1.001	0.189			
ALT	0.984	0.978–0.991	< 0.001	0.977	0.967–0.988	< 0.001
g-GT	1.003	1.001–1.006	0.003	1.003	0.999–1.007	0.091
ALP	1.003	1.002–1.004	< 0.001	1.002	1.000–1.004	0.025
ALB	0.420	0.294–0.599	< 0.001	3.068	0.378–24.893	0.292
Plt	1.000	1.000–1.000	< 0.001	1.000	1.000–1.000	0.588
Age	1.078	1.058–1.098	< 0.001	1.078	1.050–1.107	< 0.001
Sex (Male vs Female)	3.850	2.638–5.620	< 0.001	6.328	3.637–11.011	< 0.001

*Abbreviations*: *NLR* Neutrophil–Lymphocyte Ratio, *ALBI* Albumin-Bilirubin Score, *FIB4-index* Fibrosis-4 Index, *AFP* Alpha-Fetoprotein, *BMI* Body Mass Index, *AST* Aspartate Aminotransferase, *ALT* Alanine Aminotransferase, *γ-GT* Gamma-Glutamyl Transferase, *ALP* Alkaline Phosphatase, *ALB* Albumin, *Plt* Platelet, *Age* Patient Age; Sex

### The serum FOLR1 level is a prognostic biomarker of early HCC, especially in combination with the GALAD score

**Fig. 4:**
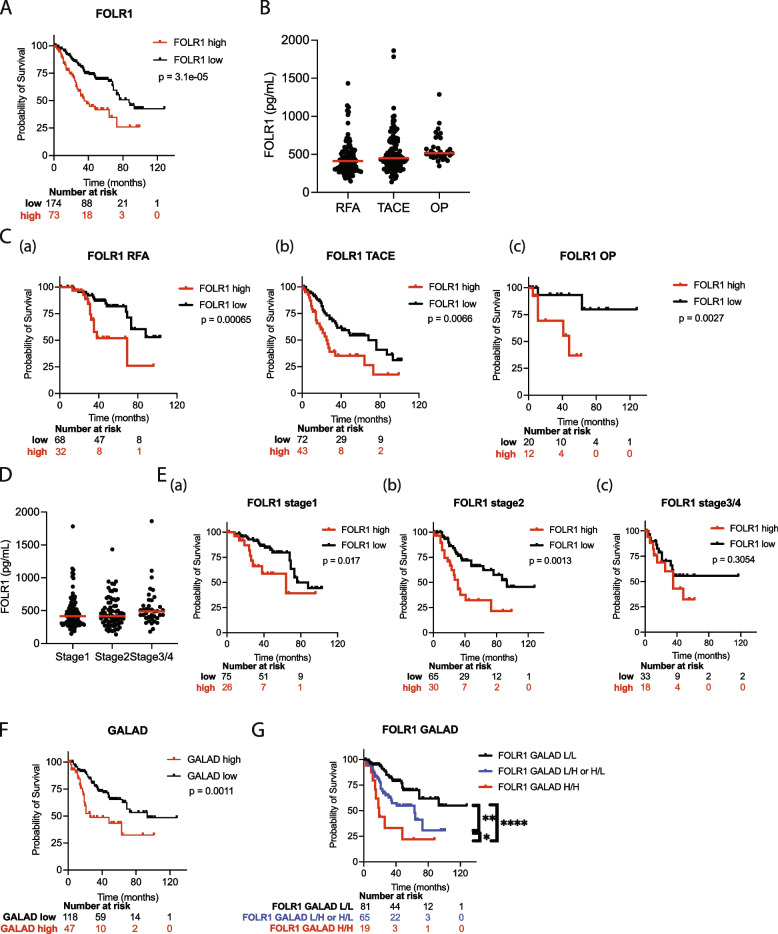
The serum FOLR1 level is a prognostic biomarker of early HCC, especially in combination with the GALAD score. **(A)** Kaplan‒Meier survival curves for HCC patients stratified by high (red line) and low (black line) serum FOLR1 levels. **(B)** Comparison of serum FOLR1 levels among patients treated with radiofrequency ablation (RFA), transarterial chemoembolization (TACE), and operation (OP). **(C)** Subgroup analysis of survival, showing survival curves for patients with high and low serum FOLR1 levels and stratified by treatment with RFA (FOLR1 RFA), TACE (FOLR1 TACE) and OP (FOLR1 OP). **(D)** Serum FOLR1 levels across different stages of HCC (stage 1 to stage 4). **(E)** Subgroup analysis of survival, showing survival curves for patients with high and low serum FOLR1 levels and stratified by HCC stage (**(a)** FOLR1 stage 1, **(b)** FOLR1 stage 2 and **(c)** FOLR1 stage 3). **(F)** Survival curves of HCC patients stratified by the GALAD score. **(G)** Survival curves of patients stratified by serum FOLR1 levels and GALAD score into the following groups: low/low (L/L), low/high or high/low (L/H or H/L), and high/high (H/H). *p < 0.05. **p < 0.01. ****p < 0.0001

Next, we evaluated the utility of serum FOLR1 levels as a prognostic marker in 247 HCC patients (Table 1). We stratified patients into two groups based on the cutoff value of serum FOLR1 levels determined by the Youden index. HCC patients with high serum FOLR1 levels experienced significantly shorter OS than those with low serum FOLR1 levels (Fig. 4A). A subgroup analysis was then conducted based on the treatment methods used for HCC. No significant differences in FOLR1 levels were observed among the 3 treatment methods, including radiofrequency ablation (RFA), transarterial chemoembolization (TACE) and operation (OP) (Fig. 4B). Patients with high serum FOLR1 levels had a significantly worse prognosis than those with low serum FOLR1 levels, regardless of treatment (Fig. 4C). A subgroup analysis based on HCC stage did not reveal significant differences in FOLR1 levels among the groups (Fig. 4D). However, patients early-stage tumors (stages 1 and 2) presenting with high FOLR1 levels had a significantly worse prognosis (Fig. 4E). Thus, the serum FOLR1 level is highly useful as a prognostic marker, especially for early-stage HCC. We then investigated the clinical factors associated with high FOLR1 expression. The high FOLR1 group presented lower hemoglobin levels and serum albumin levels and higher serum creatinine levels and FIB-4 index, ALBI, and Child‒Pugh scores than the low FOLR1 group did, but no significant differences in tumor size, number, or stage were observed (Table 3). FOLR1 levels showed a tendency to increase as the degree of differentiation decreased, but it was not statistically significant (Supplementary Fig. 4). An examination of the correlation between FOLR1 levels and the levels of various clinical markers revealed no strong associations with clinical variables (Supplementary Fig. 5). A univariate Cox proportional hazard analysis revealed that FOLR1, GALAD score, the neutrophil‒lymphocyte ratio (NLR), the FIB-4 index, age, AST levels, ALP levels, albumin levels, AFP, ALBI, stage and treatment methods were associated with a poor prognosis (Table 4). The multivariate analysis revealed that high FOLR1 levels and the GALAD score were associated with poor prognosis (Table 4). Indeed, HCC patients with high GALAD scores experienced significantly shorter OS than did those with low GALAD scores (Fig. 4F). The multivariate analysis of subgroup revealed that FOLR1 was associated with poor prognosis among patients who underwent surgical treatment and who were either stage I or II, while GALAD score was associated with poor prognosis among patients who underwent TACE treatment (Supplementary Table 2–7). Finally, we integrated these two prognostic predictors of HCC and found that the combination of FOLR1 levels and the GALAD score further stratified patients according to the prognosis (Fig. 4G). Overall, serum FOLR1 levels may be a prognostic biomarker of early HCC, especially in combination with the GALAD score.

**Table 3:** Background of HCC patients with FOLR1 high or low expression in our cohort

Factor	Unit	FOLR1-H(*n* = 74)		FOLR1-L(*n* = 173)		*P* value
		MEDIAN	IQR	MEDIAN	IQR	
FOLR1	ng/µL	708.7	614.7–852.1	387.7	305.0–507.1	< 0.001
Age	Years	77.0	70–81	74.0	67–80	0.088
Sex(M/F)		47/27		127/46		0.122
BMI	kg/m2	22.1	20.3–25.3	22.6	20.7–25.3	0.981
WBC	/mm3	4150.0	3175–5372.5	4860.0	3610–5805	0.418
Hb	g/dL	11.6	10.2–12.8	13.2	12.0–14.3	< 0.001
Plt	10^4^/μL	11.2	7.9–16.4	13.9	8.1–17.7	0.149
AST	U/L	43.5	29.8–63.5	38	30–55	0.024
ALT	U/L	27.5	20–44.3	30	20–44	0.230
ALP	U/L	384	287–531.5	296.5	234–405	0.056
Na	mmol/L	140	138.5–141	140	138–141	0.711
T-Bil	mg/dL	0.7	0.5–1	0.7	0.5–1	0.732
γ-GT	U/L	42.5	33.8–70.5	48	26.5–89	0.951
Cr	mg/dL	0.99	0.81–1.63	0.76	0.66–0.86	< 0.001
PT	%	78	67–87.9	81	70–90	0.538
PT-INR		1.13	1.07–1.20	1.1	1.04–1.17	0.403
ALB	g/dL	3.5	3.2–3.9	3.8	3.4–4.2	< 0.001
AFP	ng/mL	16	5.5–126	9	5–48	0.058
AFP-L3	ng/mL	5.95	3.5–20.38	7.25	4.1–25.68	0.889
FIB4		5.61	3.50–9.51	4.16	2.48–7.33	0.009
GALAD		3.81	2.62–7.07	4.20	2.44–6.01	0.282
NLR		2.55	1.67–3.61	2.30	1.52–3.33	0.440
Child–Pugh	(5/6/7/8/9/10)	15/16/18/7/4/1		84/34/21/11/2/2		0.001
ALBI		-2.31	-2.61—-1.93	-2.56	-2.86—-2.18	< 0.001
Tumor Diameter	mm	13.00	10–18	13.00	10–20	0.841
Tumor Number		1	1–2	1	1–2	0.153
Stage (I/II/III/IV)		26/30/15/3		75/65/30/3		0.683

*Abbreviations*: *Age* Patient Age, *Sex (M/F)* Sex (Male/Female), *BMI* Body Mass Index, *WBC* White Blood Cell count, *Hb* Hemoglobin, *Plt* Platelet, *AST* Aspartate Aminotransferase, *ALT* Alanine Aminotransferase, *ALP* Alkaline Phosphatase, *Na* Sodium, *T-Bil* Total Bilirubin, *γ-GT* Gamma-Glutamyl Transferase, *Cr* Creatinine, *PT* Prothrombin Time, *PT-INR* Prothrombin Time-International Normalized Ratio, *ALB* Albumin, *AFP* Alpha-Fetoprotein, *AFP-L3* Alpha-Fetoprotein-L3, *FIB4* Fibrosis-4 Index, *GALAD* Gender, Age, AFP-L3, AFP, DCP (a scoring system for HCC prediction), *NLR* Neutrophil–Lymphocyte Ratio, *Child–Pugh* Child–Pugh Score, *ALBI* Albumin-Bilirubin Score, *Tumor Diameter* Tumor Diameter, *Tumor Number* Number of Tumors, *Stage (I/II/III/IV)* Tumor Stage (I to IV), *IQR* Interquartile Range

**Table 4:** Cox Proportional Hazards Regression Model for Predicting Overall Survival in HCC Patients

Factor	Univariate analysis			Multivariate analysis		
	Hazard ratio	95% CI	*p* value	Hazard ratio	95% CI	*p* value
FOLR1	1.002	1.001–1.003	< 0.001	1.002	1.001–1.004	0.005
GALAD	1.208	1.108–1.310	< 0.001	1.134	1.018–1.263	0.022
NLR	1.189	1.036–1.343	0.015	1.140	0.886–1.465	0.308
FIB4	1.063	1.027–1.096	0.001	0.999	0.945–1.056	0.973
Age	1.041	1.017–1.067	0.001	1.022	0.988–1.056	0.212
BMI	0.968	0.917–1.024	0.258			
Plt	1.000	1.000–1.000	0.140			
AST	1.011	1.006–1.018	< 0.001	1.009	0.997–1.021	0.155
ALT	1.001	0.992–1.010	0.763			
ALP	1.001	1.000–1.002	0.007	0.999	0.998–1.001	0.537
ALB	0.335	0.228–0.496	< 0.001	1.215	0.138–10.695	0.861
g-GT	1.000	0.997–1.001	0.642			
AFP	1.000	1.000–1.000	0.018	1.000	1.000–1.000	0.324
ALBI	3.260	2.129–4.970	< 0.001	3.875	0.329–45.590	0.282
Sex (Male vs Female)	0.802	0.512–1.257	0.343			
Stage (II-IV vs I)	1.885	1.207–2.943	0.004	1.640	0.700–3.842	0.255
Treatment (TACE vs RFA)	2.543	1.592–4.062	< 0.001	1.794	0.779–4.131	0.148

*Abbreviations*: *GALAD* Gender, Age, AFP-L3, AFP, DCP (a scoring system for HCC prediction), *NLR* Neutrophil–Lymphocyte Ratio, *FIB4* Fibrosis-4 Index, *BMI* Body Mass Index, *Plt* Platelet, *AST* Aspartate Aminotransferase, *ALT* Alanine Aminotransferase, *ALP* Alkaline Phosphatase, *ALB* Albumin, *g-GT* Gamma-Glutamyl Transferase, *AFP* Alpha-Fetoprotein, *ALBI* Albumin-Bilirubin Score, *Stage (II-IV vs I)* Tumor Stage (II-IV versus I)

## Discussion

In this study, we identified FOLR1 as a biomarker of HCC. The physiological roles of FOLR1 include the cellular uptake of folate, which plays an important role in cell growth, differentiation, and proliferation. FOLR1 has been reported to be overexpressed in multiple cancers of epithelial origin [27], and overexpression of FOLR1 is associated with cancer progression and a poor patient prognosis [28, 29]. Additionally, FOLR1 or portions of the receptor are released into the circulation and function as serum markers for ovarian cancer [23, 30]. However, the role of FOLR1 in HCC has not been reported. This study is the first to show the potential utility of both tumor-derived and circulating FOLR1 as a prognostic biomarker for HCC. Especially, serum FOLR1 levels serve as a diagnostic marker for HCC and a prognostic indicator for early-stage disease.

Recent research has emphasized the role of cancer stem cells in driving HCC progression, metastasis, and treatment resistance [22]. Indeed, a variety of cancer stem cell and stemness markers are closely associated with the aggressive behavior and poor clinical outcomes of HCC. Therefore, in this study, we used a novel strategy to identify prognostic markers for HCC by focusing on genes strongly associated with HCC stem/progenitor markers, including KRT19, EPCAM, and PROM1. This strategy resulted in the discovery of FOLR1, which was strongly associated with a poor prognosis. FOLR1 is known to activate pathways such as the ERK [31, 32] and JAK/STAT3 [33, 34] pathways, which are critical for stem cell survival and proliferation. The uptake of folic acid is renewed in cancer cells, and folic acid is thought to contribute to cell proliferation [35]. Excessive administration of folic acid has also been reported to promote liver carcinogenesis in a rat model [36]. FOLR1 is also known to be a transcription factor that controls the expression of genes involved in stem cell maintenance, such as OCT4, SOX2, and KLF2 [37, 38]. Interestingly, our study revealed that high FOLR1 expression was associated with a variety of oncogenic pathways, including the p53, Myc, E2F, and PI3K/AKT/mTOR pathways.These reports might expect any functional role of FOLR1 in tumor aggressiveness/stemness in HCC. Meanwhile, the observed associations of FOLR1 with KRT19, EPCAM, and PROM1 in our study are only correlative and could be influenced by other factors. Future research is necessary to investigate the causal link between FOLR1, biological aggressiveness, and stem/progenitor features in HCC.

Various biomarkers have been studied, but new biomarkers other than AFP have not yet become routine in the diagnosis of HCC [39]. AFP is the most widely used serum biomarker for HCC diagnosis and HCC surveillance using ultrasound and AFP at semiannual intervals is recommended in the American Association for the Study of Liver Diseases (AASLD) guideline [40]. However, its role is debated due to limitations in sensitivity. In addition, not all HCC cases produce AFP, and its levels can also be elevated in cirrhosis or hepatitis. A large prospective study found AFP positivity (≥ 11 ng/mL) in 46% of all HCC cases and only 23.4% in small HCC (< 2 cm) [41]. Another survey indicated that nearly half of HCC patients, especially those with early or small tumors, are AFP-negative, highlighting its limitations [41]. Consequently, the European Association for the Study of the Liver (EASL) clinical practice guidelines described that the utility of AFP as biomarker is suboptimal in terms of cost-effectiveness for routine surveillance of early HCC (evidence low) [42]. This highlights the need for new biomarkers, particularly for AFP-negative HCC patients. In this study, the multivariate logistic regression analysis identified serum FOLR1 levels as an independent diagnostic factor for HCC. While the diagnostic performance of FOLR1 was comparable to that of AFP, combining these two markers significantly increased the diagnostic accuracy, suggesting potential diagnostic utility. In our cohort, ALT, ALP, age, and sex are also recognized as independent diagnostic factors for HCC in CHC patients. Age is a significant factor as prolonged liver injury increases HCC risk, and males are at higher risk due to hormonal and environmental factors, both of which are well-known risk factors of HCC [43]. While elevated ALT levels typically reflect active liver inflammation, lower ALT levels have been associated with HCC occurrence, particularly in cirrhotic patients, where reduced hepatocyte function and numbers leads to lower ALT release [44]. Elevated ALP levels may indicate cholestasis or hepatic dysfunction, both of which are linked to advanced liver disease and HCC development [45]. Including these parameters strengthens the diagnostic framework for HCC in CHC patients.

More importantly, our study revealed the potential of serum FOLR1 levels as a prognostic indicator for HCC patients. Patients with high serum FOLR1 levels experienced significantly shorter OS than those with low serum FOLR1 levels did, regardless of the therapeutic procedure. Moreover, the multivariate Cox proportional hazard analysis proved that the serum FOLR1 level independently predicted the prognosis of patients with HCC. Currently, no definitive prognostic biomarkers are available for HCC. The GALAD score was originally developed for the early detection of HCC [46] and has recently drawn attention as a prognostic marker. Several groups have shown its usefulness for predicting the prognosis of HCC [47–50]. Consistently, we also found that the GALAD score was capable of stratifying patients according to prognosis in our HCC cohort. Since both FOLR1 levels and the GALAD score are independent predictors of a poor prognosis in patients with HCC, we tested the potential of their combination and demonstrated a better stratification capacity. While the combination may improve predictive performance, it also increases model complexity, raising concerns about overfitting and reduced generalizability. Overfitting can limit the model's applicability to new datasets, emphasizing the need for robust validation methods such as cross-validation and external cohort testing. Additionally, the increased complexity may impact clinical usability, as intricate models are harder to implement in practice. Future efforts should focus on balancing improved accuracy with simplicity to ensure the model's practicality in real-world settings. In this study, serum FOLR1 levels were measured preoperatively and the potential role of postoperative FOLR1 dynamics in influencing patient outcomes remains unexplored. Future studies should investigate whether changes in FOLR1 levels after surgery correlate with prognosis, as this could provide further insights into its utility as a dynamic biomarker for monitoring treatment response and disease progression.

Our study has several important limitations. First, while the biomarker potential of tumoral FOLR1 was confirmed in the validation cohort, that of serum FOLR1 was only tested in the single retrospective cohort. A prospective validation study will be initiated. Second, our cohort for the serum analysis included only Japanese patients; thus, the robustness of our findings across races could not be evaluated. Third, we could not compare the diagnostic ability of FOLR1 and GALAD scores for HCC because of the large number of missing values of Lens culinaris agglutinin-reactive fraction of alpha-fetoprotein (AFP-L3) in CHC patients without HCC and in the colon polyp cohort. Forth, patients with colon polyps were used as the control group; however, this group may not serve as an appropriate healthy control population, as there is no information regarding the effect of colon polyps on serum FOLR1 levels. Fifth, there is no cohort of patients treated with systemic therapy. Therefore, the value of FOLR1 in predicting the prognosis of these patients remains to be elucidated.

## Conclusions

In conclusion, our study presents compelling evidence supporting the potential utility of FOLR1 as a prognostic marker for HCC, with implications for improved risk stratification and personalized treatment approaches. Further validation studies are warranted to establish the clinical utility of FOLR1 and its potential impact on HCC management.

## Supplementary Information

Supplementary Material 1: Supplementary Fig. 1. Workflow for Identifying FOLR1 as a Biomarker in HCC. Schematic of the workflow used to identify FOLR1 as a biomarker. Genes upregulated in hepatocellular carcinoma (HCC) and correlated with cancer stemness markers (KRT19, PROM1, and EPCAM) were compared to identify candidate genes. These candidates were further filtered to select secretory proteins associated with overall survival (OS). FOLR1 was validated using independent cohorts for diagnostic and prognostic efficacy (created with Biorender).

Supplementary Material 2: Supplementary Fig. 2. Correlation between the expression of the FOLR1 mRNA and stemness-related genes. Scatter plot showing the correlations between KRT19 and EPCAM **(a)**, KRT19 and PROM1 (b), and EPCAM and PROM1 (c) mRNA levels in the TIGER-LC cohort.

Supplementary Material 3: Supplementary Fig. 3. Nomogram for HCC Prediction and its Diagnostic Performance. (a) Nomogram for predicting HCC based on *FOLR1*, ALP, ALT, sex, age, and AFP. Each variable contributes points, which are summed to estimate the probability of HCC. Density plots next to each predictor illustrate their distributions in the dataset. (b) ROC curves comparing the diagnostic performance of FOLR1 (purple), AFP (green), and the combined nomogram model (black). The area under the curve (AUC) values for FOLR1, AFP, and the nomogram are 0.689, 0.709, and 0.883, respectively, indicating superior predictive performance of the nomogram.

Supplementary Material 4: Supplementary Fig. 4. Expression of *FOLR1* mRNA Across Tumor Grades. Quantitative PCR analysis showing the expression levels of FOLR1 mRNA across different tumor grades (G1–G4) in HCC samples from TCGA-LIHC cohort.

Supplementary Material 5: Supplementary Fig. 5. Correlations between FOLR1 mRNA levels and clinical markers. Scatter plot showing the correlations between FOLR1 mRNA levels and aspartate aminotransferase (AST) levels, aspartate aminotransferase (ALT) levels, platelet counts, the neutrophil–lymphocyte ratio (NLR), des-gamma-carboxy prothrombin (DCP) levels, AFP levels, the FIB4 index, albumin–bilirubin (ALBI) score and GALAD score.

Supplementary Material 6

## Data Availability

The study utilizes processed whole exome sequencing data along with publicly accessible datasets, including TCGA (TCGA-LIHC) [https://portal.gdc.cancer.gov/projects/TCGA-LIHC] and TIGER-LC data, which can be accessed through the dbGaP repository under Study Accession phs001199.v2.p1.

## Abbreviations

AFP: Alpha-fetoprotein
AFP-L3: Lens culinaris agglutinin-reactive fraction of alpha-fetoprotein
ALBI: Albumin–bilirubin
ALP: Alkaline phosphatase
ALT: Alanine aminotransferase
AST: Aspartate aminotransferase
AUROC: Area Under the Receiver Operating Characteristic curve
CHC: Chronic hepatitis C
CT: Computed tomography
DCP: Des-gamma-carboxy prothrombin
FIB-4: Fibrosis-4
FOLR1: Folate receptor 1
GALAD: Gender, Age, AFP-L3, AFP, and Des-gamma-carboxy prothrombin
ICC: Intrahepatic cholangiocarcinoma
MRI: Magnetic Resonance Imaging
NLR: Neutrophil–lymphocyte ratio
OP: Operation
OS: Overall survival
PLC: Primary liver cancer
RFA: Radiofrequency ablation
TACE: Transarterial chemoembolization
TCGA: The Cancer Genome Atlas
TIGER-LC: The Thailand Initiative in Genomics and Expression Research for Liver Cancer
