# Supplementary figures and images for: Folate receptor 1 is a stemness trait-associated diagnostic and prognostic marker for hepatocellular carcinoma
Source: Biomark Res. 2025 Mar 4;13:37. doi: 10.1186/s40364-025-00752-8 (PMC11877696; doi:10.1186/s40364-025-00752-8)

Supple Fig.1

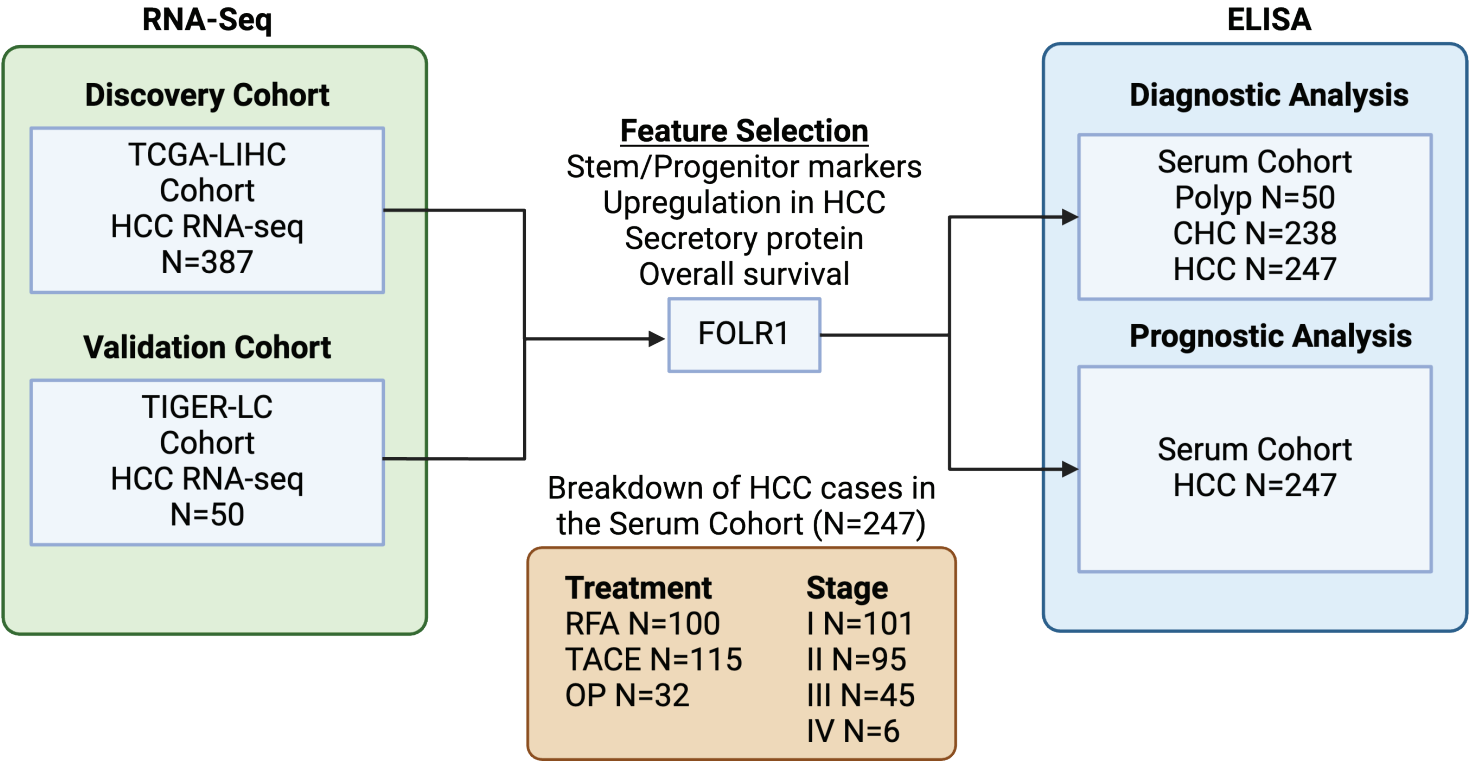

Supplement: Supplementary file 1 — Supplementary Material 1: Supplementary Fig. 1. Workflow for Identifying FOLR1 as a Biomarker in HCC. Schematic of the workflow used to identify FOLR1 as a biomarker. Genes upregulated in hepatocellular carcinoma (HCC) and correlated with cancer stemness markers (KRT19, PROM1, and EPCAM) were compared to identify candidate genes. These candidates were further filtered to select secretory proteins associated with overall survival (OS). FOLR1 was validated using independent cohorts for diagnostic and prognostic efficacy (created with Biorender). [file 40364_2025_752_MOESM1_ESM.pdf]

Supple Fig.2

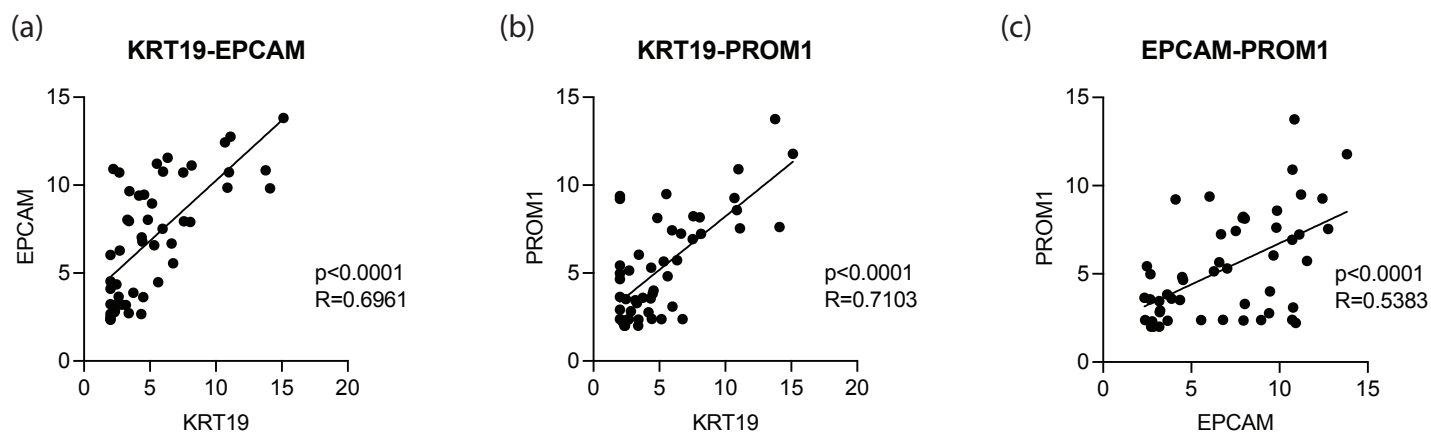

Supplement: Supplementary file 2 — Supplementary Material 2: Supplementary Fig. 2. Correlation between the expression of the FOLR1 mRNA and stemness-related genes. Scatter plot showing the correlations between KRT19 and EPCAM (a), KRT19 and PROM1 (b), and EPCAM and PROM1 (c) mRNA levels in the TIGER-LC cohort. [file 40364_2025_752_MOESM2_ESM.pdf]

Supple Fig.3

(a)

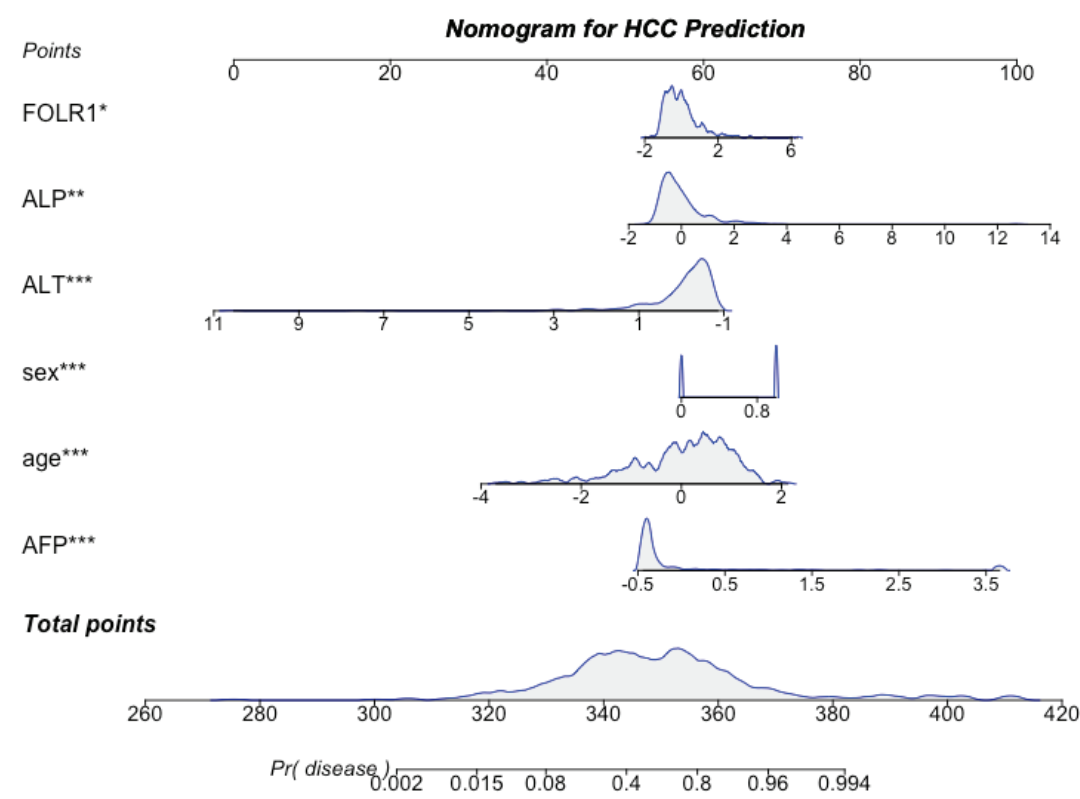

(b)

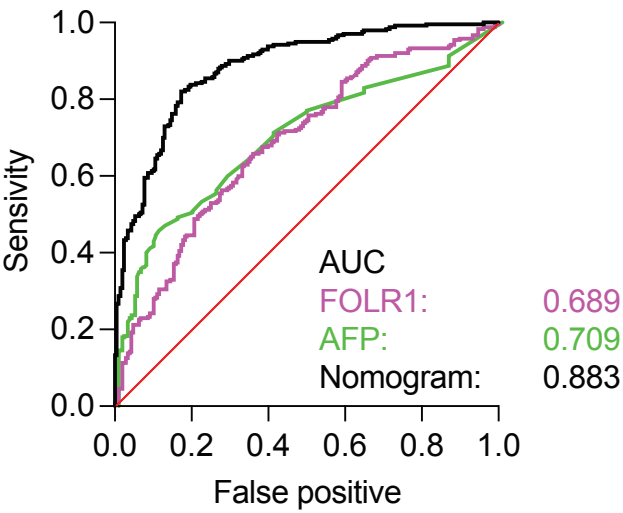

Supplement: Supplementary file 3 — Supplementary Material 3: Supplementary Fig. 3. Nomogram for HCC Prediction and its Diagnostic Performance. (a) Nomogram for predicting HCC based on FOLR1, ALP, ALT, sex, age, and AFP. Each variable contributes points, which are summed to estimate the probability of HCC. Density plots next to each predictor illustrate their distributions in the dataset. (b) ROC curves comparing the diagnostic performance of FOLR1 (purple), AFP (green), and the combined nomogram model (black). The area under the curve (AUC) values for FOLR1, AFP, and the nomogram are 0.689, 0.709, and 0.883, respectively, indicating superior predictive performance of the nomogram. [file 40364_2025_752_MOESM3_ESM.pdf]

Supple Fig.4

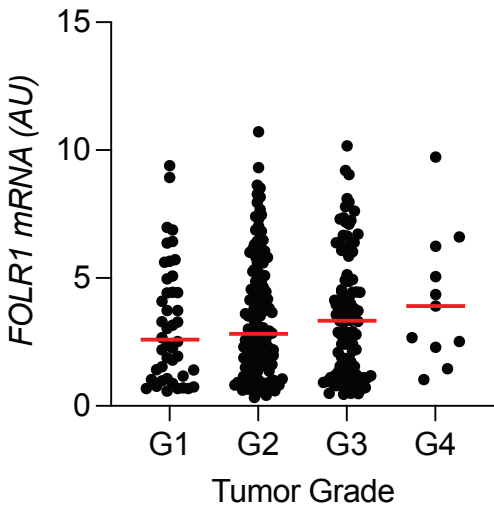

Supplement: Supplementary file 4 — Supplementary Material 4: Supplementary Fig. 4. Expression of FOLR1 mRNA Across Tumor Grades. Quantitative PCR analysis showing the expression levels of FOLR1 mRNA across different tumor grades (G1–G4) in HCC samples from TCGA-LIHC cohort. [file 40364_2025_752_MOESM4_ESM.pdf]

Supple Fig.5

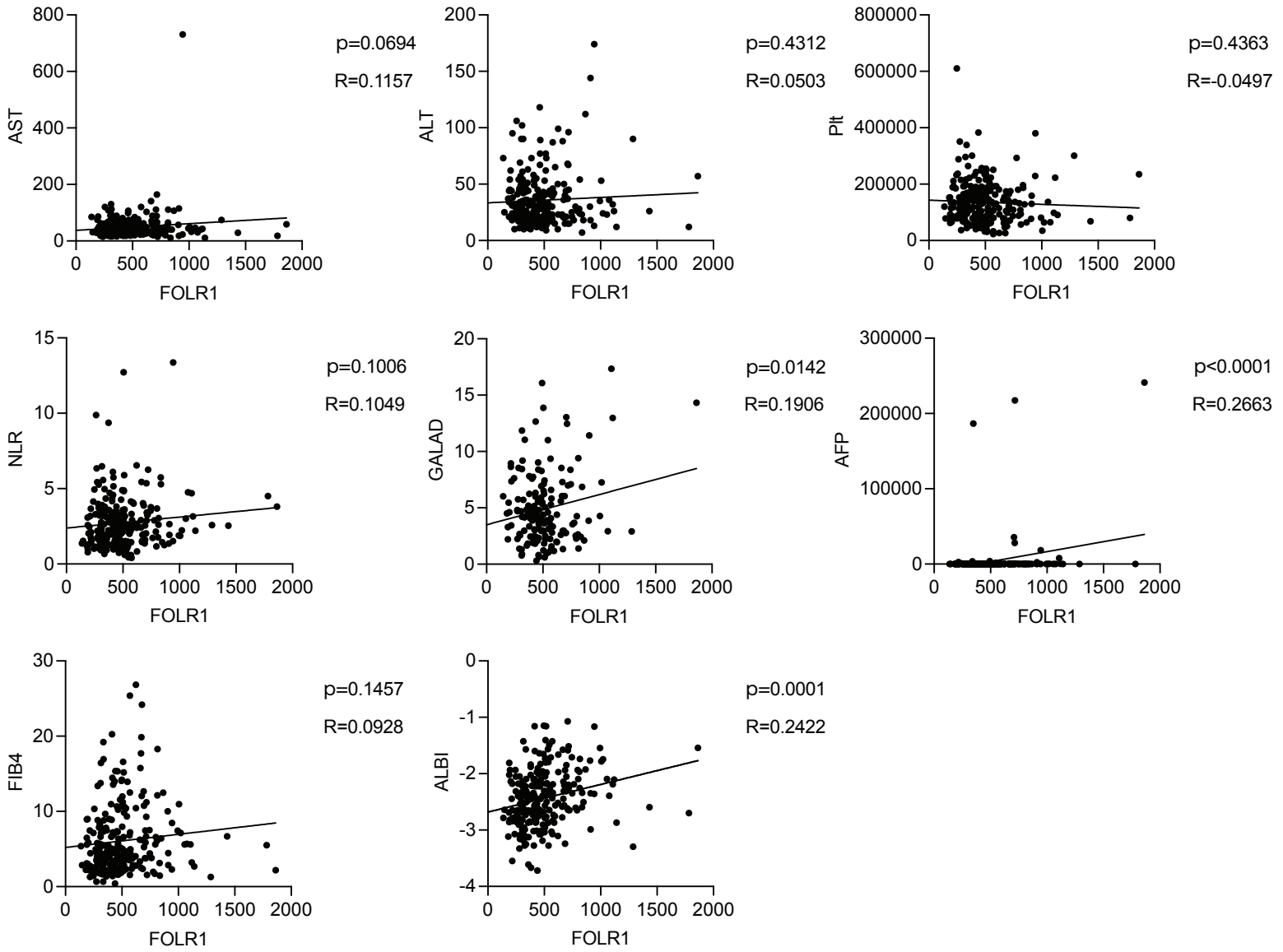

Supplement: Supplementary file 5 — Supplementary Material 5: Supplementary Fig. 5. Correlations between FOLR1 mRNA levels and clinical markers. Scatter plot showing the correlations between FOLR1 mRNA levels and aspartate aminotransferase (AST) levels, aspartate aminotransferase (ALT) levels, platelet counts, the neutrophil–lymphocyte ratio (NLR), des-gamma-carboxy prothrombin (DCP) levels, AFP levels, the FIB4 index, albumin–bilirubin (ALBI) score and GALAD score. [file 40364_2025_752_MOESM5_ESM.pdf]
