# Supplementary material for: Folate receptor 1 is a stemness trait-associated diagnostic and prognostic marker for hepatocellular carcinoma
Source: Biomark Res. 2025 Mar 4;13:37. doi: 10.1186/s40364-025-00752-8 (PMC11877696; doi:10.1186/s40364-025-00752-8)
Supplement: Supplementary file 6 — Supplementary Material 6 [file 40364_2025_752_MOESM6_ESM.docx]

**Supplementary Table 1. Correlation Coefficients of 25 Candidate Biomarkers with Stemness Markers and Hazard Ratios for Overall Survival in HCC**

| Gene Name | Spearman correlation coefficient | | | Hazard Ratio | 95% CI | p Value |
| --- | --- | --- | --- | --- | --- | --- |
|  | KRT19 | EPCAM | PROM1 |  |  |  |
| ADAM12 | 0.496 | 0.379 | 0.449 | 1.067 | 0.988-1.153 | 0.100 |
| BMP8A | 0.403 | 0.425 | 0.381 | 1.004 | 0.902-1.117 | 0.946 |
| COL11A1 | 0.510 | 0.369 | 0.510 | 1.050 | 0.983-1.122 | 0.146 |
| COL1A1 | 0.494 | 0.414 | 0.464 | 1.030 | 0.951-1.115 | 0.474 |
| COL22A1 | 0.442 | 0.380 | 0.414 | 1.011 | 0.953-1.073 | 0.716 |
| COL4A2 | 0.400 | 0.351 | 0.366 | 1.009 | 0.893-1.140 | 0.883 |
| COL9A2 | 0.589 | 0.499 | 0.574 | 1.022 | 0.941-1.111 | 0.601 |
| COMP | 0.486 | 0.398 | 0.445 | 1.011 | 0.955-1.071 | 0.703 |
| CPXM1 | 0.464 | 0.394 | 0.412 | 1.033 | 0.955-1.118 | 0.421 |
| CST2 | 0.466 | 0.411 | 0.421 | 1.024 | 0.935-1.121 | 0.612 |
| CTHRC1 | 0.478 | 0.416 | 0.425 | 1.041 | 0.972-1.114 | 0.248 |
| FOLR1 | 0.479 | 0.362 | 0.426 | 1.073 | 1.005-1.146 | 0.035 |
| GDF10 | 0.382 | 0.409 | 0.411 | 1.065 | 0.994-1.141 | 0.075 |
| LGI2 | 0.443 | 0.360 | 0.432 | 1.043 | 0.944-1.154 | 0.408 |
| LTBP2 | 0.397 | 0.393 | 0.409 | 0.963 | 0.875-1.060 | 0.440 |
| MFAP2 | 0.580 | 0.540 | 0.556 | 1.026 | 0.951-1.106 | 0.506 |
| MMP11 | 0.511 | 0.532 | 0.460 | 0.987 | 0.920-1.058 | 0.706 |
| NPTX1 | 0.486 | 0.434 | 0.472 | 1.065 | 0.970-1.168 | 0.186 |
| PCSK1N | 0.467 | 0.469 | 0.404 | 1.018 | 0.966-1.072 | 0.504 |
| PCYOX1L | 0.427 | 0.424 | 0.372 | 1.007 | 0.858-1.181 | 0.936 |
| PKDCC | 0.442 | 0.482 | 0.369 | 1.013 | 0.898-1.142 | 0.835 |
| PTK7 | 0.447 | 0.540 | 0.450 | 1.028 | 0.941-1.122 | 0.542 |
| SCG2 | 0.456 | 0.372 | 0.467 | 1.012 | 0.929-1.104 | 0.779 |
| SCUBE3 | 0.377 | 0.363 | 0.377 | 0.963 | 0.834-1.112 | 0.611 |
| TNFSF15 | 0.432 | 0.439 | 0.434 | 1.027 | 0.927-1.137 | 0.610 |
|  |  |  |  |  |  |  |
| Abbreviations: T, tumor; NT, non-tumor | | | |  |  |  |

**Supplementary Table 2. Cox Proportional Hazards Model for Predicting Overall Survival in HCC Patients Undergoing RFA Treatment**

| Factor | Univariate analysis | | | Multivariate analysis | | |
| --- | --- | --- | --- | --- | --- | --- |
|  | Hazard ratio | 95% CI | *p* value | Hazard ratio | 95% CI | *p* value |
| FOLR1 | 1.003 | 1.001-1.005 | 0.013 | 1.002 | 0.999-1.004 | 0.279 |
| GALAD | 1.144 | 0.822-1.518 | 0.403 |  |  |  |
| NLR | 1.260 | 0.921-1.704 | 0.146 |  |  |  |
| FIB4 | 3.181 | 1.548-6.350 | 0.002 | 1.435 | 0.035-48.264 | 0.845 |
| Age | 1.095 | 1.020-1.163 | 0.015 | 1.035 | 0.945-1.126 | 0.451 |
| BMI | 1.000 | 0.990-1.001 | 0.499 |  |  |  |
| Plt | 0.988 | 0.894-1.106 | 0.817 |  |  |  |
| AST | 1.003 | 0.986-1.018 | 0.733 |  |  |  |
| ALT | 0.989 | 0.965-1.008 | 0.257 |  |  |  |
| ALP | 1.001 | 0.998-1.002 | 0.721 |  |  |  |
| ALB | 1.000 | 0.997-1.002 | 0.470 |  |  |  |
| g-GT | 0.359 | 0.195-0.688 | 0.003 | 0.636 | 0.024-13.819 | 0.779 |
| AFP | 1.000 | 1.000-1.000 | 0.063 |  |  |  |
| ALBI | 1.077 | 1.026-1.137 | 0.002 | 1.064 | 1.016-1.121 | 0.008 |
| Sex (Male vs Female) | 0.942 | 2.119-0.419 | 0.885 |  |  |  |
| Stage (II-IV vs I) | 3.241 | 1.436-7.318 | 0.008 | 3.332 | 1.373-8.088 | 0.011 |

Abbreviations: GALAD, Gender, Age, AFP-L3, AFP, DCP (a scoring system for HCC prediction); NLR, Neutrophil-Lymphocyte Ratio; FIB4, Fibrosis-4 Index; BMI, Body Mass Index; Plt, Platelet; AST, Aspartate Aminotransferase; ALT, Alanine Aminotransferase; ALP, Alkaline Phosphatase; ALB; Albumin, g-GT, Gamma-Glutamyl Transferase; AFP, Alpha-Fetoprotein; ALBI, Albumin-Bilirubin Score; Stage (II-IV vs I), Tumor Stage (II-IV versus I)

**Supplementary Table 3. Cox Proportional Hazards Model for Predicting Overall Survival in HCC Patients Undergoing TACE Treatment**

| Factor | Univariate analysis | | | Multivariate analysis | | |
| --- | --- | --- | --- | --- | --- | --- |
|  | Hazard ratio | 95% CI | *p* value | Hazard ratio | 95% CI | *p* value |
| FOLR1 | 1.002 | 1.001-1.003 | 0.001 | 1.002 | 1.000-1.004 | 0.087 |
| GALAD | 1.212 | 1.089-1.338 | 0.001 | 1.251 | 1.114-1.404 | 0.001 |
| NLR | 1.114 | 0.957-1.269 | 0.156 |  |  |  |
| FIB4 | 2.576 | 1.379-4.828 | 0.003 | 5.264 | 0.334-86.285 | 0.239 |
| Age | 1.035 | 0.990-1.076 | 0.128 |  |  |  |
| BMI | 1.001 | 1.000-1.001 | 0.251 |  |  |  |
| Plt | 0.952 | 0.882-1.021 | 0.174 |  |  |  |
| AST | 1.010 | 1.005-1.018 | 0.002 | 1.011 | 0.997-1.024 | 0.138 |
| ALT | 0.998 | 0.985-1.010 | 0.724 |  |  |  |
| ALP | 0.998 | 0.992-1.003 | 0.446 |  |  |  |
| ALB | 1.001 | 1.001-1.002 | 0.012 | 1.000 | 0.998-1.002 | 0.773 |
| g-GT | 0.359 | 0.189-0.666 | 0.002 | 2.207 | 0.177-27.258 | 0.538 |
| AFP | 1.000 | 1.000-1.001 | 0.997 |  |  |  |
| ALBI | 1.038 | 1.009-1.069 | 0.013 | 1.015 | 0.978-1.055 | 0.441 |
| Sex (Male vs Female) | 0.725 | 0.407-1.292 | 0.285 |  |  |  |
| Stage (II-IV vs I) | 0.992 | 0.532-1.849 | 0.978 |  |  |  |

Abbreviations: GALAD, Gender, Age, AFP-L3, AFP, DCP (a scoring system for HCC prediction); NLR, Neutrophil-Lymphocyte Ratio; FIB4, Fibrosis-4 Index; BMI, Body Mass Index; Plt, Platelet; AST, Aspartate Aminotransferase; ALT, Alanine Aminotransferase; ALP, Alkaline Phosphatase; ALB; Albumin, g-GT, Gamma-Glutamyl Transferase; AFP, Alpha-Fetoprotein; ALBI, Albumin-Bilirubin Score; Stage (II-IV vs I), Tumor Stage (II-IV versus I)

**Supplementary Table 4. Cox Proportional Hazards Model for Predicting Overall Survival in HCC Patients Undergoing OP Treatment**

| Factor | Univariate analysis | | | | Multivariate analysis | | | |
| --- | --- | --- | --- | --- | --- | --- | --- | --- |
|  | Hazard ratio | 95% CI | *p* value | Hazard ratio | | 95% CI | *p* value |  |
| FOLR1 | 1.006 | 1.003-1.009 | 0.002 | 1.021 | | 1.008-1.039 | <0.001 |  |
| GALAD | 1.113 | 0.885-1.142 | 0.308 |  | |  |  |  |
| NLR | 1.793 | 0.741-4.127 | 0.187 |  | |  |  |  |
| FIB4 | 5.488 | 0.446-68.846 | 0.184 |  | |  |  |  |
| Age | 1.603 | 0.923-2.825 | 0.089 |  | |  |  |  |
| BMI | 1.001 | 1.001-1.002 | 0.007 | 1.003 | | 1.001-1.007 | <0.001 |  |
| Plt | 0.918 | 0.716-1.191 | 0.504 |  | |  |  |  |
| AST | 1.027 | 1.006-1.048 | 0.017 | 0.858 | | 0.812-0.962 | 0.005 |  |
| ALT | 1.017 | 0.998-1.033 | 0.087 |  | |  |  |  |
| ALP | 1.000 | 0.993-1.003 | 0.695 |  | |  |  |  |
| ALB | 1.000 | 0.995-1.003 | 0.722 |  | |  |  |  |
| g-GT | 0.291 | 0.027-3.058 | 0.300 |  | |  |  |  |
| AFP | 1.001 | 1.000-1.001 | 0.697 |  | |  |  |  |
| ALBI | 0.954 | 0.878-1.043 | 0.281 |  | |  |  |  |
| Sex (Male vs Female) | 0.591 | 0.108-3.243 | 0.558 |  | |  |  |  |

Abbreviations: GALAD, Gender, Age, AFP-L3, AFP, DCP (a scoring system for HCC prediction); NLR, Neutrophil-Lymphocyte Ratio; FIB4, Fibrosis-4 Index; BMI, Body Mass Index; Plt, Platelet; AST, Aspartate Aminotransferase; ALT, Alanine Aminotransferase; ALP, Alkaline Phosphatase; ALB; Albumin, g-GT, Gamma-Glutamyl Transferase; AFP, Alpha-Fetoprotein; ALBI, Albumin-Bilirubin Score

**Supplementary Table 5. Cox Proportional Hazards Model for Predicting Overall Survival in Stage I HCC Patients**

| Factor | Univariate analysis | | | Multivariate analysis | | |
| --- | --- | --- | --- | --- | --- | --- |
|  | Hazard ratio | 95% CI | *p* value | Hazard ratio | 95% CI | *p* value |
| FOLR1 | 1.002 | 1.001-1.003 | 0.005 | 1.002 | 1.001-1.004 | 0.023 |
| GALAD | 1.117 | 0.824-1.444 | 0.454 |  |  |  |
| NLR | 0.994 | 0.738-1.281 | 0.962 |  |  |  |
| FIB4 | 3.736 | 1.808-7.551 | 0.001 | 2.786 | 0.118-61.832 | 0.521 |
| Age | 1.162 | 1.084-1.240 | <0.001 | 1.164 | 1.027-1.300 | 0.020 |
| BMI | 1.000 | 0.992-1.001 | 0.512 |  |  |  |
| Plt | 1.283 | 0.567-2.903 | 0.544 |  |  |  |
| AST | 1.108 | 0.998-1.023 | 0.103 |  |  |  |
| ALT | 1.000 | 0.981-1.015 | 0.917 |  |  |  |
| ALP | 1.000 | 0.997-1.002 | 0.897 |  |  |  |
| ALB | 1.002 | 0.999-1.004 | 0.305 |  |  |  |
| g-GT | 0.329 | 0.180-0.626 | 0.001 | 1.482 | 0.096-20.957 | 0.775 |
| AFP | 1.000 | 1.000-1.000 | 0.007 | 1.001 | 1.000-1.001 | 0.383 |
| ALBI | 1.090 | 1.038-1.151 | 0.001 | 1.047 | 0.996-1.107 | 0.076 |
| Sex (Male vs Female) | 1.283 | 0.567-2.903 | 0.544 |  |  |  |

Abbreviations: GALAD, Gender, Age, AFP-L3, AFP, DCP (a scoring system for HCC prediction); NLR, Neutrophil-Lymphocyte Ratio; FIB4, Fibrosis-4 Index; BMI, Body Mass Index; Plt, Platelet; AST, Aspartate Aminotransferase; ALT, Alanine Aminotransferase; ALP, Alkaline Phosphatase; ALB; Albumin, g-GT, Gamma-Glutamyl Transferase; AFP, Alpha-Fetoprotein; ALBI, Albumin-Bilirubin Score

**Supplementary Table 6. Cox Proportional Hazards Model for Predicting Overall Survival in Stage II HCC Patients**

| Factor | Univariate analysis | | | Multivariate analysis | | |
| --- | --- | --- | --- | --- | --- | --- |
|  | Hazard ratio | 95% CI | *p* value | Hazard ratio | 95% CI | *p* value |
| FOLR1 | 1.003 | 1.002-1.004 | 0.001 | 1.003 | 1.001-1.004 | 0.002 |
| GALAD | 1.123 | 0.958-1.307 | 0.151 |  |  |  |
| NLR | 1.166 | 0.941-1.392 | 0.152 |  |  |  |
| FIB4 | 3.011 | 1.406-6.492 | 0.005 | 2.041 | 0.114-30.929 | 0.620 |
| Age | 1.031 | 0.978-1.076 | 0.244 |  |  |  |
| BMI | 1.001 | 1.000-1.001 | 0.794 |  |  |  |
| Plt | 0.977 | 0.913-1.055 | 0.529 |  |  |  |
| AST | 1.013 | 1.005-1.025 | 0.001 | 1.011 | 1.003-1.024 | 0.005 |
| ALT | 1.009 | 0.992-1.024 | 0.297 |  |  |  |
| ALP | 0.996 | 0.989-1.001 | 0.095 |  |  |  |
| ALB | 1.001 | 1.000-1.002 | 0.051 |  |  |  |
| g-GT | 0.356 | 0.166-0.745 | 0.006 | 0.856 | 0.058-11.089 | 0.908 |
| AFP | 1.000 | 1.000-1.001 | 0.994 |  |  |  |
| ALBI | 1.037 | 1.002-1.074 | 0.040 | 1.042 | 1.003-1.083 | 0.035 |
| Sex (Male vs Female) | 0.532 | 0.271-1.054 | 0.082 |  |  |  |

Abbreviations: GALAD, Gender, Age, AFP-L3, AFP, DCP (a scoring system for HCC prediction); NLR, Neutrophil-Lymphocyte Ratio; FIB4, Fibrosis-4 Index; BMI, Body Mass Index; Plt, Platelet; AST, Aspartate Aminotransferase; ALT, Alanine Aminotransferase; ALP, Alkaline Phosphatase; ALB; Albumin, g-GT, Gamma-Glutamyl Transferase; AFP, Alpha-Fetoprotein; ALBI, Albumin-Bilirubin Score

**Supplementary Table 7. Cox Proportional Hazards Model for Predicting Overall Survival in Stage III and IV HCC Patients**

| Factor | Univariate analysis | | | Multivariate analysis | | |
| --- | --- | --- | --- | --- | --- | --- |
|  | Hazard ratio | 95% CI | *p* value | Hazard ratio | 95% CI | *p* value |
| FOLR1 | 1.003 | 1.000-1.005 | 0.052 |  |  |  |
| GALAD | 1.211 | 1.078-1.357 | 0.002 | 1.181 | 0.996-1.397 | 0.056 |
| NLR | 1.630 | 1.195-2.203 | 0.003 | 1.239 | 0.811-1.857 | 0.311 |
| FIB4 | 2.491 | 1.223-5.451 | 0.026 | 4.199 | 1.265-15.569 | 0.019 |
| Age | 1.047 | 0.951-1.141 | 0.332 |  |  |  |
| BMI | 1.001 | 1.001-1.001 | 0.037 | 1.001 | 1.000-1.001 | 0.319 |
| Plt | 0.968 | 0.868-1.067 | 0.517 |  |  |  |
| AST | 1.009 | 0.992-1.023 | 0.307 |  |  |  |
| ALT | 0.996 | 0.976-1.010 | 0.570 |  |  |  |
| ALP | 1.002 | 0.997-1.006 | 0.430 |  |  |  |
| ALB | 1.001 | 1.000-1.003 | 0.348 |  |  |  |
| g-GT | 0.311 | 0.133-0.710 | 0.006 |  |  |  |
| AFP | 1.000 | 1.000-1.001 | 0.780 |  |  |  |
| ALBI | 1.012 | 0.962-1.066 | 0.671 |  |  |  |
| Sex (Male vs Female) | 0.823 | 0.332-2.045 | 0.678 |  |  |  |

Abbreviations: GALAD, Gender, Age, AFP-L3, AFP, DCP (a scoring system for HCC prediction); NLR, Neutrophil-Lymphocyte Ratio; FIB4, Fibrosis-4 Index; BMI, Body Mass Index; Plt, Platelet; AST, Aspartate Aminotransferase; ALT, Alanine Aminotransferase; ALP, Alkaline Phosphatase; ALB; Albumin, g-GT, Gamma-Glutamyl Transferase; AFP, Alpha-Fetoprotein; ALBI, Albumin-Bilirubin Score
